# Selecting Day-to-Day Functional Task Assessments Amenable to Video Telehealth for Persons with Dementia

**DOI:** 10.1093/geroni/igaf122.2872

**Published:** 2025-12-31

**Authors:** Megan Gately, Jaye McLaren, Alexandra Howard, Steven Shirk, Helen Hoenig, Lauren Moo

**Affiliations:** VA Bedford Health Care System, Bedford, Massachusetts, United States; VA Bedford Healthcare System, Bedford, Massachusetts, United States; VA Bedford Health Care System, Bedford, Massachusetts, United States; VA Bedford Health Care System, Bedford, Massachusetts, United States; Durham VA Health Care System, Durham, North Carolina, United States; VA Bedford Healthcare System, Bedford, Massachusetts, United States

## Abstract

Assessment of day-to-day function is critical to prevent losses due to dementia, with observation of a day-to-day task such as toothbrushing being the gold standard. Patients with dementia (PWD) face barriers to such an assessment. In-home video telehealth may increase access, but the feasibility of its use to observe PWD completing day-to-day tasks has not been demonstrated. This project describes the process of selecting specific functional task assessments feasible for adaptation to video telehealth delivery to PWD. We sent ten subject matter experts (SMEs) from fields of occupational therapy, social work, geriatrics, neuropsychology, and nursing a list of 20 day-to-day tasks, such as toothbrushing and medication management, sourced from standardized and non-standardized tools. SMEs rated the feasibility of assessing tasks over video using 5-point Likert scales on domains of clinical importance, privacy and safety concerns, time to administer, and overall feasibility. The most feasible tasks for video administration were pouring a drink, washing hands, cleaning a counter, and toothbrushing. The least feasible tasks were meal preparation, tub/shower mobility, and toilet mobility. The tasks with the highest perceived clinical importance were medication management, tub/shower mobility, and home safety. Tasks with the most privacy and safety concerns involved more intimate activities: tub/shower mobility, toilet mobility, and dressing. Determining the feasibility of video assessment of day-to-day tasks involves weighing several complex factors. Understanding clinician perspectives of which tasks are amenable to video assessment will facilitate the adaptation of assessment processes to remote administration for PWD, thereby increasing access to care.

